# Effect of gluten free diet on immune response to gliadin in patients with non-celiac gluten sensitivity

**DOI:** 10.1186/1471-230X-14-26

**Published:** 2014-02-13

**Authors:** Giacomo Caio, Umberto Volta, Francesco Tovoli, Roberto De Giorgio

**Affiliations:** 1 Department of Medical and Surgical Sciences, St. Orsola-Malpighi Hospital, University of Bologna, Via Massarenti, 9, 40138 Bologna, Italy; 2 Department of Digestive Diseases and Internal Medicine, St. Orsola-Malpighi Hospital, University of Bologna, Via Massarenti, 9, 40138 Bologna, Italy; 3 Centro di Ricerca Bio-Medica Applicata (C.R.B.A.), St. Orsola-Malpighi Hospital, University of Bologna, Bologna, Italy

**Keywords:** Anti gliadin antibodies, Non-celiac gluten sensitivity, Celiac disease

## Abstract

**Background:**

Non-celiac gluten sensitivity is a syndrome characterized by gastrointestinal and extra-intestinal symptoms occurring in a few hours/days after gluten and/or other wheat protein ingestion and rapidly improving after exclusion of potential dietary triggers. There are no established laboratory markers for non-celiac gluten sensitivity, although a high prevalence of first generation anti-gliadin antibodies of IgG class has been reported in this condition. This study was designed to characterize the effect of the gluten-free diet on anti-gliadin antibodies of IgG class in patients with non-celiac gluten sensitivity.

**Methods:**

Anti-gliadin antibodies of both IgG and IgA classes were assayed by ELISA in 44 non-celiac gluten sensitivity and 40 celiac disease patients after 6 months of gluten-free diet.

**Results:**

The majority of non-celiac gluten sensitivity patients (93.2%) showed the disappearance of anti-gliadin antibodies of IgG class after 6 months of gluten-free diet; in contrast, 16/40 (40%) of celiac patients displayed the persistence of these antibodies after gluten withdrawal. In non-celiac gluten sensitivity patients anti-gliadin antibodies IgG persistence after gluten withdrawal was significantly correlated with the low compliance to gluten-free diet and a mild clinical response.

**Conclusions:**

Anti-gliadin antibodies of the IgG class disappear in patients with non-celiac gluten sensitivity reflecting a strict compliance to the gluten-free diet and a good clinical response to gluten withdrawal.

## Background

The spectrum of gluten-related disorders has recently acquired a new entity represented by non-celiac gluten sensitivity (NCGS) [1-3]. This is an emerging syndrome evoked by gluten ingestion in patients in whom both celiac disease (CD) and wheat allergy have been excluded [4-6]. In addition to gluten, other triggers involved in NCGS pathogenesis have been recently identified including wheat proteins (i.e. amylase- and trypsin- inhibitors) [7] and fermentable oligo-, di-, mono-saccharides and polyols (FODMAPs) [8,9]. NCGS is characterized by gastro-esophageal reflux disease (i.e., retrosternal pyrosis and regurgitation) and irritable bowel syndrome (IBS) like symptoms (i.e., abdominal pain, bloating, diarrhea, constipation and alternating bowel) along with extra-intestinal manifestations (“foggy mind”, headache, fatigue, joint and muscle pain, leg/arm numbness, eczema/rash, depression/anxiety and anemia) that occur soon after gluten ingestion, rapidly improving after gluten withdrawal and relapsing in a few hours or days after gluten challenge [2]. NCGS and CD seems to be different because of epidemiologic and pathogenetic aspects. NCGS is thought to be more frequent than CD, although its actual prevalence is still poorly defined [3,10]; CD recognizes pathogenic mechanisms which have been defined over the years, while the mechanisms underlying NCGS remain largely unsettled [2]. Furthermore, patients diagnosed as NCGS must prove to be negative for anti-endomysial (EmA) and anti-tissue transglutaminase antibodies (tTGA) and have no or mild changes matching Marsh 0/1 of the small intestinal mucosa [9]. Specific IgE and/or prick tests should be tested in order to exclude wheat allergy [6]. A double-blind, placebo controlled challenge is suggested to confirm diagnosis, since no biomarker is so far available for establishing a firm diagnosis of NCGS [11,12].

Serology has been of paramount importance for CD diagnosis, thus we postulate that antibody screening might help to detect at least a subset of NCGS patients. For more than 20 years, prior to highly specific tests (i.e., EmA and tTGA), the detection of anti-gliadin antibodies (AGA) has been instrumental to stratify patients with suspected CD. Their positivity was a diagnostic criterion for endoscopic evaluation confirming CD in the majority of cases [13]. However, the usefulness of this test has been hampered by false-positive cases ranging from 5 to 20%, especially for IgG, rather than IgA, AGA [14]. Nonetheless, we have recently shown that 56% and 8% of NCGS patients had IgG and IgA AGA positivity, respectively [15], findings in line with results shown by others [3,12].

Our study was designed to evaluate the effect of gluten withdrawal on AGA detected in the serum of NCGS patients. Moreover, we investigated whether a correlation exists between AGA persistence and compliance to gluten-free diet (GFD) as well as AGA persistence and clinical response to GFD.

## Methods

### Patients

We studied 44 cases of NCGS (female/male 28/16, median age 38 years - range 17 to 63 years), all positive for AGA IgG (only 4 were positive also for AGA IgA), selected from a series of 78 NCGS patients analyzed in a previously published paper [15]. The diagnosis of NCGS was established after a thorough work-up in our CD outpatient clinic (a tertiary referral center for CD of the Emilia-Romagna Region in Italy at the Department of Medical and Surgical Sciences at St. Orsola-Malpighi Hospital) between January 2009 and June 2011. All the 44 patients included in the present study were negative for CD serology (EmA and tTGA of IgA class) and for wheat allergy tests (specific IgE and skin prick tests) on a gluten containing diet. All these patients were referred to our attention because of intestinal and extraintestinal symptoms with an early onset (a few hours or days) after gluten ingestion. Small intestinal biopsy, tested in all of them on a gluten-containing diet, showed either a normal mucosa (Marsh 0) (n = 26) (58%) or mild abnormalities (n = 18) (42%), with an increased number of intra-epithelial lymphocytes (Marsh 1) [16].

A serum sample was collected from the 44 NCGS patients after 6 months of GFD and was reassessed for AGA IgG and IgA. The persistence of AGA was correlated with dietary adherence and clinical response to GFD. Dietary adherence to the GFD was assessed in all NCGS patients through a structured questionnaire, which was conducted by a dietician with experience in educating patients with gluten-related disorders. Patients were classified as strict compliers (people who after the beginning of GFD had never ingested gluten knowingly) and low compliers (those who admitted frequent dietary lapses). The clinical response of NCGS patients to GFD was evaluated on the basis of the persistence or disappearance of the gastrointestinal (pyrosis, regurgitation, bloating, abdominal pain, diarrhoea, constipation, alternating bowel) and extra-intestinal symptoms (“foggy mind”, headache, fatigue, joint and muscle pain, leg/arm numbness, eczema/rash, depression/anxiety and anemia) scored from 0 to 3 as follows: 0 = absent; 1 = occasionally present, without worsening quality of life; 2 = frequently present, with a moderate worsening of quality of life; 3 = always present, with a severe worsening of quality of life. Patients with NCGS were classified as “good responder” to GFD when all the symptoms experienced on a gluten containing diet disappeared or significantly improved after GFD with a decrease of the initial global score higher than 50%. Those showing a decrease of the initial global score lower than 50% were classified as “mild responder”.

The immune response to gliadin after 6 months of GFD was also evaluated in 40 age- and sex-matched CD patients, all positive for AGA IgG when untreated (of whom 30 were positive for AGA IgA). The diagnosis of CD had been confirmed in the 40 CD patients included in the present study by the demonstration of villous atrophy and the positivity for EmA IgA and tTGA IgA on a gluten containing diet. AGA persistence in CD patients after gluten withdrawal was correlated with dietary adherence and clinical response to GFD, by using the same approach adopted for NCGS patients.

As no individual patient identification was involved and all assays where part of clinical routine practice, a simplified International Review Board approval by the St. Orsola-Malpighi Hospital Ethics Committee was obtained.

### Serological tests

AGA IgG and IgA were determined in NCGS and CD patients by commercially available kits of enzyme-linked immunosorbent assay (ELISA) (α-gliatest SIgG and SIgA, Eurospital, Trieste, Italy) using purified α-gliadin as antigen. The cut-off levels, as suggested by the manufacturer, were fixed at 50 arbitrary units (AU) for IgG and 15 AU for IgA [17].

### Statistical analysis

The two-tailed Fisher’s exact test was used to evaluate the correlation between AGA persistence and the compliance with GFD as well as the clinical improvement after gluten withdrawal in both NCGS and CD patients.

## Results

**Figure 1:**
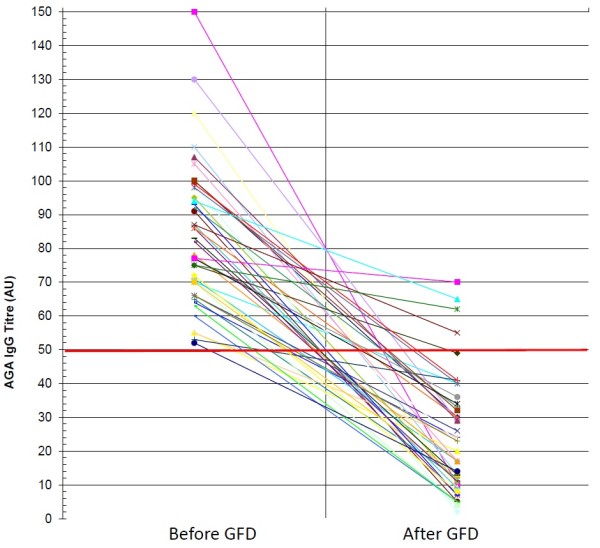
**IgG antigliadin antibodies before and after GFD in NCGS patients: anti-gliadin antibodies (AGA) of IgG class before and after gluten free diet (GFD) in patients with non-celiac gluten sensitivity (NCGS).** Only three of the 44 patients studied showed persistence of AGA IgG at a low titre after gluten withdrawal.

**Figure 2:**
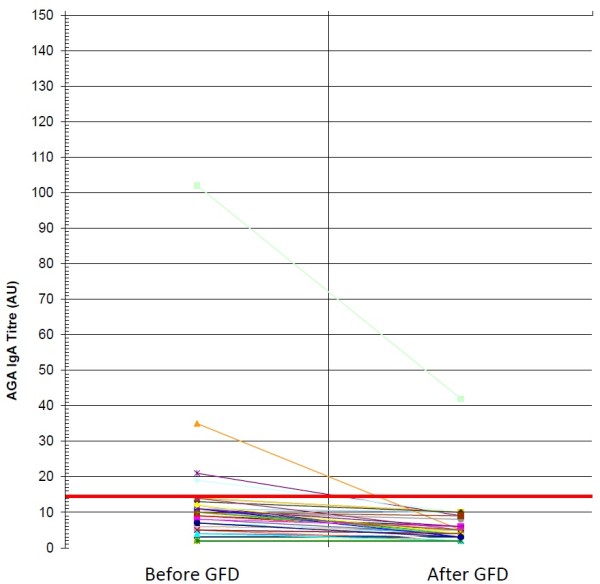
IgA antigliadin antibodies before and after GFD in NCGS patients: anti-gliadin antibodies (AGA) of IgA class before and after gluten free diet (GFD) in patients with non-celiac gluten sensitivity (NCGS).

AGA IgG persisted positive only in 3 (6.8%) out of the 44 NCGS patients tested after 6 months of gluten withdrawal (Figure 1). AGA IgG persistence after GFD was significantly correlated with the low degree of compliance with gluten-free diet and with a mild clinical response (*P* = 0.009 and *P* = 0.00075, two-tailed Fisher’s exact test, respectively) (Table 1). AGA IgG disappeared in 40 out of the 41 NCGS patients who followed a strict GFD and remained positive in 2 out of the 3 low compliers admitting frequently dietary lapses. Of the 39 NCGS patients classified as good responders to the diet on the basis of the disappearance or significant improvement of intestinal and extra-intestinal symptoms, none maintained the positivity for AGA IgG. On the contrary, these antibodies were still detected in 3 out of the 5 patients assessed as mild responders to gluten withdrawal due to a partial resolution of clinical symptoms. In the CD group the persistence of AGA IgG after 6 months of GFD did not show any correlation either with the compliance to the diet or the clinical response following the gluten-free regimen (Figure 2). AGA IgG persistence was detected in 13 (40.6%) out of the 32 strict compliers and in 3 (37.5%) out of the 8 low compliers. There was no significant difference for AGA IgG persistence in treated CD between good (43.3%) and mild responders (30%).

**Table 1:** AGA IgG in NCGS and CD related to compliance to the GFD and clinical picture

**44 NCGS patients (all with AGA IgG when untreated) after 6-months-GFD**	**Good response to GFD 39 /44**		**Mild response to GFD 5/44**	
Compliance	AGA IgG +	AGA IgG-	AGA IgG +	AGA IgG-
Strict 41/44	0	38	1	2
Low 3/44	0	1	2	0
**40 CD patients (all with AGA IgG on a gluten containing diet) after 6-months-GFD**	**Good response to GFD 30/40**		**Mild response to GFD 10/40**	
Compliance	AGA IgG+	AGA IgG-	AGA IgG +	AGA IgG-
Strict 32/40	11	14	2	5
Low 8/40	2	3	1	2

AGA IgG in NCGS patients with low compliance vs. AGA IgG in NCGS patients with strict compliance, P = 0.009; AGA IgG in NCGS patients with mild response to GFD vs IgG AGA in NCGS patients with good response to GFD. NCGS, P = 0.00075. Two tailed Fisher’s exact test.

AGA IgG in CD patients with low compliance vs. AGA IgG in CD patients with strict compliance, P = NS; AGA IgG in CD patients with mild response to GFD vs AGA IgG in CD patients with good response to GFD, P = NS. Two tailed Fisher’s exact test.

**Figure 3:**
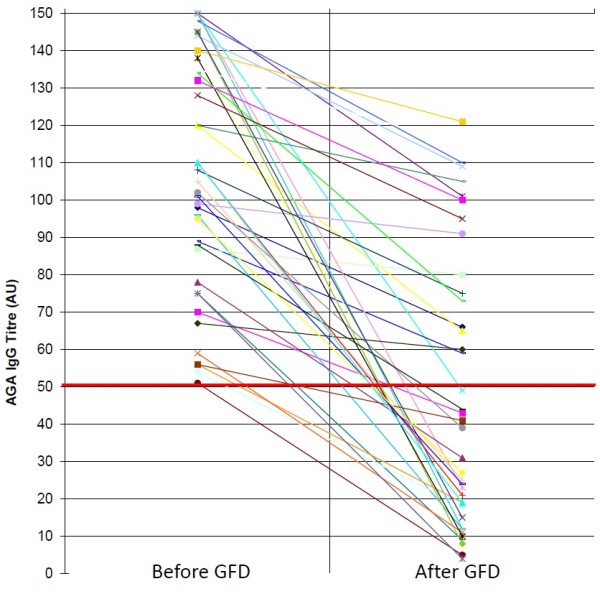
IgG antigliadin antibodies before and after GFD in CD patients: anti-gliadin antibodies (AGA) of IgG class before and after gluten free diet (GFD) in patients with celiac disease (CD).

**Figure 4:**
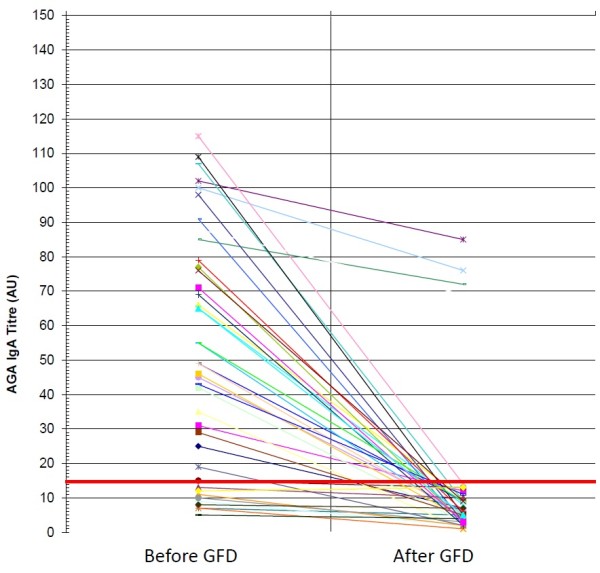
IgA antigliadin antibodies before and after GFD in CD patients: anti-gliadin antibodies (AGA) of IgA class before and after gluten free diet (GFD) in patients with celiac disease (CD).

AGA IgA were positive in only 4 of the 44 NCGS patients on gluten-containing diet. These antibodies remained positive in one patient who admitted a low compliance to GFD and had a mild clinical response. In the other 3 NCGS patients, who adhered strictly to GFD and had a very good clinical impact on symptoms, the AGA IgA became negative (Figure 3). In the celiac group the persistence of AGA IgA after gluten withdrawal was strictly related to the low compliance with the diet and with the mild clinical response to the dietary treatment (*P* = 0.000036 and *P* = 00018, two-tailed Fisher’s exact test, respectively) (Figure 4). Only 4 out of the 30 AGA IgA positive CD patients showed the persistence of these antibodies; all of them showed a mild clinical response and were not complying with the GFD.

## Discussion

Despite the progressive awareness of its existence, NCGS is still a condition with many unanswered questions. In contrast to CD, the prevalence of NCGS is far from being established since few reliable epidemiologic studies have been so far published [3,10]. Indeed, the National Health and Nutrition Examination Survey has identified 49 cases of NCGS over 7,762 subjects (age range 6-80 years) in the 2009-2010 period with a weighted prevalence of 0.55% [10]. In a tertiary care center for celiac research, the criteria for NCGS were met by 347 over 5,896 patients observed between 2004 and 2010 with a prevalence of 5.9% [3]. This latter figure should be cautiously taken as a patient selection bias may have occurred. In our last year (2012) experience at the Celiac Disease Centre of St. Orsola-Malpighi Hospital in Bologna, the ratio between the new NCGS and CD cases was 1.6 to 1, confirming a slightly higher prevalence of NCGS vs. CD [9].

A further aspect of distinction between CD and NCGS is given by the pathogenetic mechanisms underlying these two diseases. Indeed, while both adaptive and innate immunity are well known to have a major role in CD, only innate immunity has been thought to be activated by gluten proteins in NCGS [3]. However, even adaptive immunity may play a role in NCGS as recently suggested [18]. In support of this possibility, previous papers have shown that antibodies, such as AGA, can be detected in more than half of NCGS patients [3,9,12,15]. Although AGA cannot be considered a specific marker for NCGS (as these antibodies can be found in many other conditions including autoimmune disorders and even in healthy people), their positivity in the presence of gluten-related symptoms can be helpful for confirming a diagnosis of NCGS [3,12,15,19]. However, there are no data concerning the effect of the GFD on AGA in NCGS and whether AGA persistence after GFD correlates with a low compliance to gluten-withdrawal and clinical manifestations in this new gluten-related syndrome. In this context, our study provides new findings showing that GFD can improve significantly both gastrointestinal and extra-intestinal symptoms in patients with NCGS. A possible link between gluten and symptoms has been suggested in NCGS patients [11], although other components, mainly contained in the wheat (i.e. proteins), may trigger symptoms in NCGS. For example, wheat amylase- and trypsin-inhibitors, a complex of proteins triggering innate immunity, could contribute to symptom generation in NCGS [7]. Similarly to IBS, it is likely that FODMAPs may also play a role in evoking gastrointestinal (as well as extra-intestinal) symptoms in patients with NCGS [8,20]. In this line, recent evidence by Bisierkieski et al. showed that a diet low in FODMAPs resulted in an improvement of the clinical picture of NCGS in IBS patients, thus supporting a major role of these dietary factors, rather than gluten [21].

In a previous series of untreated NCGS patients we demonstrated that about 50% of cases resulted positive for AGA IgG [15]. Those patients were followed up and retested for AGA in the present study where we highlighted that NCGS and CD have a different immune response to gliadin after GFD. Interestingly, concerning NCGS, the detection of AGA IgG identifies a possible subgroup of such patients. The vast majority of NCGS patients showed AGA IgG disappearance after gluten withdrawal, whereas the same antibodies persisted in 40% of CD patients after GFD. In NCGS patients the negativization of AGA IgG after gluten withdrawal was significantly related to the strict compliance to the diet as demonstrated by the antibody persistence in only one out of the 41 patients with a strong adherence to the diet and in two of the 3 who did not adhere strictly to GFD. Moreover, AGA IgG disappearance in treated NCGS patients was closely related to the good response to GFD with a significant improvement of the clinical picture (i.e., symptom score decreased more than a half compared to that of gluten-containing diet). None of the 39 NCGS patients classified as good responders to the diet maintained AGA IgG positivity, whereas these antibodies persisted in 3 out of the 5 NCGS patients assessed as mild responders to the diet. In contrast to NCGS, AGA IgG in celiac patients persisted after GFD regardless the adherence and the clinical response to gluten withdrawal. The different behaviour of AGA IgG identified in patients with CD vs. NCGS in response to GFD may reflect the different pathogenic mechanisms underlying these two disorders. In fact, CD is a well-recognized autoimmune disease, whereas NCGS is likely a gluten hypersensitivity without an established involvement of autoimmunity [2,5]. The persistence of AGA IgG in a large proportion of CD patients following GFD can be regarded as an expression of the immunological memory of the autoimmune disorder, whereas the gluten withdrawal in NCGS switches off the immune process and this effect is supposed to lead to the rapid disappearance of AGA [22]. In contrast with AGA IgG, AGA IgA disappearance can be viewed as the result of a strict GFD as well as of a good clinical response in both NCGS and CD patients. Indeed, these antibodies disappeared in all the NCGS patients with a strict adherence to GFD and a good clinical response, remaining positive only in one patient with admitted low dietary compliance and mild clinical response to the diet. Similarly, the finding of AGA IgA in the celiac group on GFD was closely related to the low compliance with the diet and the mild clinical response to the dietary treatment. Our study focused on a specific subset of NCGS patients, i.e. those with AGA IgG positivity and whether GFD may have an effect on NCGS-related symptoms in the "antibody-negative" subset of gluten sensitive patients remains unknown. It is tentative to speculate that even in AGA-negative NCGS patients gluten withdrawal can improve the symptom profile. Clearly, further studies testing this hypothesis are eagerly awaited. The fluctuation of AGA with dietary changes (i.e. gluten withdrawal and re-challenge) in NCGS patients remains another very interesting aspect. In a recent double blind, placebo controlled study, Biesiekierski et al. have shown that NCGS patients in gluten-withdrawal for 6 weeks and re-challenged with high dose of gluten (16 g/day for one week) had an increase of AGA IgG and IgA in 8% and 21% of cases, respectively [21]. These are lower proportions of NCGS patients compared to those detected in patients during a gluten containing diet. Thus, the data provided by Biesiekierski et al. appear quite different from our results, indicating about 50% of NCGS patients with positive AGA IgG. A possible explanation for such discrepancy may be the very short period (one week) of gluten challenge used by Biesiekierski et al. [21]. Probably, this short-time gluten exposure in NCGS patients was not enough to evoke the reactivation of the adaptive immunity to produce AGA in response to gliadin. This exciting possibility should deserve further study.

## Conclusions

In conclusion, AGA IgG disappearance in patients with NCGS can be viewed as a sign of strict compliance to the GFD and an expression of a good clinical response to gluten withdrawal. The AGA IgG persistence in NCGS indicates that gluten is still introduced in the diet, and this prevents clinical improvement.

## Abbreviations

AGA: Anti-gliadin antibodies; AU: Arbitrary units; CD: Celiac disease; ELISA: Enzyme-linked immunosorbent assay; EmA: Anti-endomysial antibodies; FODMAPs: Fermentable oligo-, di-, mono-saccharides and polyols; GFD: Gluten-free diet; IBS: Irritable bowel syndrome; NCGS: Non celiac gluten sensitivity; tTGA: Anti-tissue transglutaminase antibodies.

## Competing interests

The authors declare that they have no competing interests.

## Authors’ contributions

GC: Clinical evaluation of patients, serological assay and data analysis and contributed to writing the manuscript. UV: Study design, clinical evaluation of patients, data analysis, writing the manuscript. FT: Clinical evaluation of patients, serological assay and data analysis. RDG: Data analysis and writing the manuscript and final supervision. All authors read and approved the final manuscript.

## Pre-publication history

The pre-publication history for this paper can be accessed here:

http://www.biomedcentral.com/1471-230X/14/26/prepub
